# Multiplicative synergistic risk of hepatocellular carcinoma development among hepatitis B and C co-infected subjects in HBV endemic area: a community-based cohort study

**DOI:** 10.1186/1471-2407-12-452

**Published:** 2012-10-05

**Authors:** Jin-Kyoung Oh, Hai-Rim Shin, Min Kyung Lim, Heeyoun Cho, Dong-Il Kim, Youngmee Jee, Haesun Yun, Keun-Young Yoo

**Affiliations:** 1 National Cancer Control Institute, National Cancer Center, Goyang, Republic of Korea; 2 Non Communicable Diseases and Health Promotion, World Health Organization Western Pacific Regional Office, 1000 UN Avenue, Manila, Philippines; 3 Department of Occupational and Environmental Medicine, Samsung Medical Center, Sungkunkwan University School of Medicine, Suwon, Republic of Korea; 4 Expanded Programme on Immunization, World Health Organization Western Pacific Regional Office, Manila, Philippines; 5 Division of Enteric and Hepatitis Viruses, National Institute of Health, Korea Centers for Disease Control and Prevention, Osong, Republic of Korea; 6 Department of Preventive Medicine, Seoul National University College of Medicine, Seoul, Republic of Korea

**Keywords:** Hepatitis B virus, Hepatitis C virus, Hepatocellular carcinoma, Cohort study

## Abstract

**Background:**

There has been limited study on the effect of infection with different hepatitis C virus (HCV) genotypes on the risk of hepatocellular carcinoma (HCC) in hepatitis B virus (HBV) endemic regions of Asia.

**Methods:**

Hazard ratios of HCC development were estimated for HBV and HCV co-infected subjects among a community-based prospective cohort. HCV genotype was determined in HCV RNA-positive samples. Incident HCC cases were identified through linkage to the cancer registry.

**Results:**

HCC incidence was 79 per 100,000 person-years in the study population (50 incident cases among 6,694 individuals within 63,170 person-years with an average of 9.4 years of follow-up); seroprevalence of HBsAg and anti-HCV was 5.2% and 5.6%. Adjusted hazard ratios of HCC by HBsAg positivity and anti-HCV positivity were 13.3 (CI: 7.3-24.4) and 6.7 (CI: 3.6-12.6). HRs of HBV and HCV monoinfection, and HBV/HCV coinfection were 17.1 (CI: 8.4-34.8), 10.4 (CI: 4.9-22.1) and 115.0 (CI: 32.5-407.3). Multiplicative synergistic effect of HBV/HCV coinfection on HCC risk was also observed (synergy index: 4.5, CI: 1.3-15.5). Infection with HCV genotype 1 (HR: 29.7, CI: 13.6-46.8) and mixed infection with genotype 1 and 2 (HR: 68.7, CI: 16.4-288.4) significantly elevated HCC risk, much higher than HBV infection.

**Conclusions:**

The effect of differences in HCV genotype and the multiplicative synergistic effect of HBV/HCV coinfection on HCC risk shown in the present study underline the need for comprehensive identification of hepatitis infection status in order to prevent and control HCC in this HBV endemic area.

## Background

Epidemiologic and experimental evidence has shown that chronic infection with hepatitis B and C virus (HBV, HCV) is a major risk factor for hepatocellular carcinoma (HCC) [1,2]. The highest incidence rates of HCC come from Southeast Asia including China and the Republic of Korea, and sub-Saharan Africa, where HCC is frequently caused by HBV infection, and from Japan, where HCC is predominantly caused by HCV [3]. HBV and HCV infection increase HCC risk some 12- to 14-fold and this risk differs according to whether the infection is an HBV or HCV monoinfection, or an HBV/HCV coinfection, and depends on which genotypes of the viruses are involved, and their viral load [4-9].

In particular, distribution of HCV genotypes varies according to geographic regions, which raises several issues related to their transmission and treatment. There were not so many studies on risk difference in HCV genotypes in general population. Some studies, including prospective cohort and meta-analysis, have suggested that HCV genotype 1b plays the most important role in HCC development [8,10,11] but most studies included in the meta-analysis were based on patients with chronic hepatitis or liver cirrhosis [8].

In Korea, where the incidence rate of HCC is high (24.5 per 100,000 [12]), the prevalence of HCV (1.3% [13]) infection is lower than that of HBV (3.2% [14]). A recent meta-analysis of Korean data showed that the pooled relative risk (RR) (11.5 for both sexes) of HCC among individuals positive for antibodies against HCV (anti-HCV) was also lower than that among individuals positive for hepatitis B surface antigen (HBsAg) (24.4 in men and 33.7 in women) [15]. HCV genotypes 1b and 2a were reported to be the most prevalent among six known HCV genotypes and at least 30 subtypes [13], whereas genotype C was predominant (99%) among the HBV genotypes [16-18]. An additive effect of HBV/HCV coinfection, as well as a differing risk conferred by different HCV genotypes on HCC development, were suggested by a few epidemiologic studies, including a recent meta-analysis [5]. However, there are a very limited number of studies on the impact of HCV infection on HCC development in Korea. Indeed, all of the studies included in the aforementioned meta-analysis were case–control studies, the sample sizes of which were relatively small.

In this context, using a community-based prospective cohort design, the present study investigated HCC risk in relation to HBV infection, HCV infection and other risk factors, and evaluated the difference in risk between HBV or HCV monoinfection and HBV/HCV coinfection in the Republic of Korea. HCC risk according to HCV genotype was also investigated.

## Methods

### Ethics statement

The study protocol was approved by the Institutional Review Boards of the Seoul National University Hospital and the National Cancer Center of Korea. All study participants signed an informed consent form before inclusion into their respective cohort.

### Study participants

A community-based prospective cohort study was conducted in a rural area of Korea between 1993 and 2003. Briefly, the cohort was designed to investigate the relationship between environmental exposures, lifestyle factors, host factors and the risk of cancer in Korea. Members of the cohort aged 30 years or older who were residents of Haman (county) in Kyeongsangnam-do (province), a rural area in the Southeastern part of the country, with the highest reported HCC incidence, were included in the present study. After excluding 105 cohort members with existing cancer at enrollment, 28 cohort members who developed incident cancer within 6 months of enrollment (considering asymptomatic period and late detection of cancer), and 51 with incident non-Hodgkin lymphoma and cholangiocarcinoma (as these cancers are also caused by HCV infection) [19], 6 694 cancer-free members from the cohort were included in the final analysis.

### Data collection

Cohort members completed a questionnaire and provided a blood/urine sample during a health examination at the time of enrollment into the study. Detailed data collection was described previously [20,21].

Socio-demographics, tobacco smoking and alcohol drinking, HBV and HCV infection status, results of liver function test (alanine aminotransferase [ALT] and aspartate aminotransferase [AST]), fasting blood sugar (FBS), and history of acupuncture/transfusion were included in this analysis. Alcohol consumption was categorized as follows: no drinking (0 g/day), moderate drinking (0.1-23.9 g/day) and heavy drinking (≥24.0 g/day) [22]. Body mass index (BMI) was calculated from direct measures of height and weight and categorized according to the World Health Organization standard for Asians as follows: underweight (<18.5 kg/m^2^), normal (18.5-22.9 kg/m^2^), overweight (23.0-24.9 kg/m^2^), and obese (≥25.0 kg/m^2^) [23].

### HBV and HCV testing

Enzyme immunoassay (AxSYM, Abbott Laboratories, Abbott Park, Illinois, USA) was used to determine HBV and HCV infection. Samples were tested for seropositivity to HBsAg, antibodies against hepatitis B surface antigen (anti-HBs) and anti-HCV for all study participants. HCV RNA viremia was confirmed by nested reverse transcript-polymerase chain reaction or by nucleic acid testing (NAT) (COBAS AMPLICOR HCV MONITOR test, version 2.0, Roche Molecular Systems, Branchburg, New Jersey, USA) in anti-HCV-positive samples. HCV genotypes were determined by the Okamoto’s method [24] or by line-probe assay (VERSANT HCV genotype assay (LiPA), Innogenetics, Ghent, Belgium) in HCV RNA-positive samples.

### Cancer ascertainment

On December 31, 2008, 50 HCC cases were identified in the study population through computerized record linkage to the Korea National Cancer Incidence Database of the Korean Central Cancer Registry, a very reliable registry showing 95% completeness [12].

### Statistical analysis

The Cox proportional hazards model was used to estimate the adjusted hazard ratios and corresponding 95% confidence intervals (CIs) in order to assess the independent contribution of each risk factor to the development of HCC, using SAS statistical software version 9.1 (SAS Institute, Inc., North Carolina, USA). The adjusted population-attributable fraction and the 95% CIs of HCC associated with HBV or HCV infection in the study population was estimated using STATA software version 10.0 (StataCorp, Texas, USA). The synergistic effect of HBV/HCV coinfection was computed by applying the synergy index (SI) proposed by Rothman [25,26].

## Results

The demographic characteristics of the cohort are shown in Table 1. Tobacco smoking, alcohol drinking, obesity and history of acupuncture were very common (28.9%, 31.3%, 29.5% and 49.1%, respectively) in the study population, whereas history of blood transfusion was relatively rare (4.1%). The seroprevalence of HBsAg, anti-HBs and anti-HCV was 5.2%, 56.4% and 5.6%, respectively (Table 1).

**Table 1:** General characteristics of 6,694 study participants in Korea, 1993-2003

**Characteristic**		**Male N (%)**	**Female N (%)**
Gender		2682	4012
Age (years)	<50	698(26.0)	1090(40.6)
	50-59	829(30.9)	1238(46.2)
	60-69	845(31.5)	1240(46.2)
	≥70	310(11.6)	444(16.6)
Marital status	Single/Divorced	51(1.9)	39(1.5)
	Married	2138(79.7)	2422(90.3)
	Widowed	105(3.9)	922(34.4)
	Unknown	388(14.5)	629(23.5)
Education	Under middle school	1029(38.4)	2490(92.8)
	Upper middle school	1634(60.9)	1493(55.7)
	Unknown	19(0.7)	29(1.1)
Job	Farmer	1598(59.6)	2136(79.6)
	Other	1084(40.4)	1876(69.9)
	Unknown	455(17.0)	717(26.7)
Smoking	Non-smoker	554(20.7)	3587(133.7)
	Ex-smoker	521(19.4)	66(2.5)
	Current smoker	1596(59.5)	337(12.6)
	Unknown	11(0.4)	22(0.8)
Alcohol drinking	Non-drinker	813(30.3)	3437(128.2)
	Ex-drinker	246(9.2)	66(2.5)
	Current drinker	1604(59.8)	470(17.5)
	Unknown	19(0.7)	39(1.5)
BMI (Kg/m^2^)	<23	1392(51.9)	1648(61.4)
	23-25	644(24)	942(35.1)
	≥25	591(22)	1347(50.2)
	Unknown	55(2.1)	75(2.8)
Acupuncture history	No	1192(44.4)	1408(52.5)
	Yes	1183(44.1)	2103(78.4)
	Unknown	307(11.4)	501(18.7)
Blood transfusion history	No	1890(70.5)	2744(102.3)
	Yes	85(3.2)	191(7.1)
	Unknown	707(26.4)	1077(40.2)
HBsAg	Negative	6113(227.9)	2437(90.9)
	Positive	349(13)	154(5.7)
	Unknown	232(8.7)	91(3.4)
Anti-HBs	Negative	1151(42.9)	1652(61.6)
	Positive	1422(53)	2200(82)
	Unknown	109(4.1)	160(6)
Anti-HCV	Negative	2456(91.6)	3627(135.2)
	Positive	133(5)	242(9)
	Unknown	93(3.5)	143(5.3)
FBS (mg.dL)	<100mg/dL	1173(43.7)	1865(69.5)
	100mg/dL-126mg/dL	458(17.1)	744(27.7)
	>=126mg/dL	256(9.5)	330(12.3)
	Unknown	795(29.6)	1073(40)
ALT (U/L)	<30U/L	1799(67.1)	3284(122.4)
	≥30U/L	756(28.2)	594(22.1)
	Unknown	127(4.7)	134(5)
AST (U/L)	<35U/L	1794(66.9)	3338(124.5)
	≥35U/L	759(28.3)	535(19.9)
	Unknown	129(4.8)	139(5.2)

SD, standard deviation; BMI, body mass index; HBsAg, hepatitis B surface antigen; anti-HBs, antibodies against hepatitis B surface antigen; anti-HCV, antibodies against hepatitis C virus; FBS, fasting blood sugar; ALT, alanine aminotransferase; AST, aspartate aminotransferase.

The incidence of HCC in the study population was 79.2 per 100,000 person-years ([34 men and 16 women] 50 incident cases among 6,694 individuals within 63,170 person-years with an average of 9.4 years of follow-up).

Table 2 shows the HRs and corresponding 95% CIs of HCC according to selected risk factors. Adjusted HRs of developing HCC by HBsAg positivity and by anti-HCV positivity were 13.3 (95% CI=7.3-24.4) and 6.7 (95% CI=3.6-12.6), respectively. Higher AST (≥30u/L) and ALT (≥35u/L) levels were significantly related to increased HCC risk. However the other potential risk factors except heavy alcohol drinking (aHR=2.2, 95% CI=1.1-4.4) were not significantly related to HCC risk in the multivariate model.

**Table 2:** Relative risks (RRs) and 95% confidence intervals (CIs) of hepatocellular carcinoma (HCC) according to selected characteristics

		**Person-years**	**No. of HCC**	**Crude RR (95% CI)**	**Adjusted HR**^**a**^**(95% CI)**	**Adjusted HR**^**b**^**(95% CI)**
HBsAg						
	Negative	57,319	28	1.0 (reference)	1.0 (reference)	1.0 (reference)
	Positive	3,115	18	12.0 (6.6-21.7)	12.5 (6.9-22.7)	13.3 (7.3-24.4)
	Unknown	2,737	4	2.7 (0.9-7.8)	2.4 (0.8-7.0)	0.1 (0.01-0.5)
Anti-HCV						
	Negative	56,516	31	1.0 (reference)	1.0 (reference)	1.0 (reference)
	Positive	3,880	15	6.9 (3.7-12.7)	7.0 (3.8-13.1)	6.7 (3.6-12.6)
	Unknown	2,774	4	2.5 (0.9-7.0)	2.3 (0.8-6.5)	0.1 (0.01-0.4)
FBS (mg/dL)						
	<100	26,930	15	1.0 (reference)	1.0 (reference)	
	100-126	11,737	11	1.6 (0.7-3.5)	1.6 (0.7-3.4)	
	≥126	5,746	6	1.8 (0.7-4.6)	1.6 (0.6-4.1)	
	Unknown	18,757	18	1.7 (0.9-3.4)	1.7(0.9-3.4)	
AST (U/L)						
	<35	48,247	11	1.0 (reference)	1.0 (reference)	
	≥35	11,890	36	13.5 (6.9-26.6)	11.3 (5.7-22.5)	
	Unknown	3,033	3	3.8 (1.0-13.8)	3.6 (0.99-13.2)	
ALT (U/L)						
	<30	47,841	15	1.0 (reference)	1.0 (reference)	
	≥30	12,374	32	8.5 (4.6-15.7)	8.3 (4.4-15.6)	
	Unknown	2,955	3	2.8 (0.8-9.9)	2.8 (0.8-9.9)	
Alcohol drinking (g/day)						
	0-23.9	51,193	30	1.0 (reference)	1.0 (reference)	
	≥24	7,214	16	3.8 (2.1-7.0)	2.2 (1.1-4.4)	
	Unknown	4,763	4	1.4 (0.5-4.0)	1.0 (0.3-2.8)	
Tobacco smoking						
	Never smoker	40,375	20	1.0 (reference)	1.0 (reference)	
	Past smoker	4,826	7	3.1 (1.3-7.3)	1.2 (0.4-3.3)	
	Current smoker	17,638	23	2.7 (1.5-4.8)	1.3 (0.6-2.6)	
	Unknown	331	0	-	-	
Body mass index (kg/m^2^)						
	<18.5	2,463	2	0.8 (0.2-3.5)	0.72(0.17-3.06)	
	18.5-22.9	25,634	25	1.0 (reference)	1.0 (reference)	
	23.0-24.9	15,241	8	0.5 (0.2-1.2)	0.6 (0.3-1.4)	
	≥25.0	18,780	13	0.7 (0.4-1.4)	0.96 (0.5-1.9)	
	Unknown	1,052	2	2.1 (0.5-8.7)	2.2 (0.5-9.5)	
History of acupuncture						
	Never	27,378	27	1.0 (reference)	1.0 (reference)	
	Ever	27,998	20	0.8 (0.4-1.4)	0.8 (0.5-1.5)	
	Unknown	7,794	3	0.4 (0.1-1.3)	0.5 (0.2-1.7)	
History of transfusion						
	No	40,673	31	1.0 (reference)	1.0 (reference)	
	Yes	2,132	2	1.3 (0.3-5.4)	1.5 (0.4-6.4)	
	Unknown	20,365	17	0.98 (0.5-1.8)	1.1 (0.6-2.0)	

HBsAg, hepatitis B surface antigen; anti-HCV, antibodies against hepatitis C virus; FBS, fasting blood sugar; ALT, alanine aminotransferase; AST, aspartate aminotransferase. ^a^Age- and sex-adjusted. ^b^Age-, sex- and HBsAg/anti-HCV positivity-adjusted.

There were 14 HBV/HCV coinfected study participants, of which 3 were HCC cases. Age- and sex-adjusted HRs of HBV monoinfection, HCV monoinfection and HBV/HCV coinfection for HCC were 17.1 (95% CI=8.4-34.8), 10.4 (95% CI=4.9-22.1) and 115.0 (95% CI=32.5-407.3), respectively. The synergistic effect of HBV/HCV coinfection was more than additive and was statistically significant (SI=4.5, 95% CI=1.3-15.5) (Table 3).

**Table 3:** Hazard Ratios (HRs) and 95% confidence intervals (CIs) of hepatocellular carcinoma (HCC) according to hepatitis B and C virus infection status

**Infection status**	**No. of subjects**	**Person-years**	**No. of HCC**	**HR**^**a**^**(95% CI)**
HBsAg(−) / anti-HCV(−)	5744	53,504	16	1.0 (reference)
HBsAg(+) / anti-HCV(−)	335	2,981	15	17.1 (8.4-34.8)
HBsAg(−) / anti-HCV(+)	360	3,731	12	10.4 (4.9-22.1)
HBsAg(+) / anti-HCV(+)	14	133	3	115.0 (32.5-407.3)
				SI and 95% CI: 4.5 (1.3-15.5)^b^

HBsAg, hepatitis B surface antigen; anti-HCV, antibodies against hepatitis C virus. ^a^HR: age- and sex-adjusted. ^b^SI: synergy index=(RR_11_-1)/(RR_01_+RR_10_-2), in which RR_11_=relative risk of the joint effect of two risk factors; RR_01_ and RR_10_=relative risk of each risk factor in the absence of the other. Two hundred and forty-one subjects including 4 HCC cases had no information on HBsAg or anti-HCV, and were excluded from the analysis.

Additional file 1: Table S1 shows the distribution of HCV genotypes among 142 HCV RNA-positive subjects in the study population. As shown in Table 4, HCV genotype 1 (mostly 1b) significantly elevated HCC risk (adjusted HR=29.7, 95% CI=13.6-46.8), whereas genotype 2 did not. HR of HCC in individuals multiply infected with HCV genotype 1 and 2 (adjusted HR=68.7, 95% CI=16.4-288.4) was much higher than in monotypic infection with genotype 1 but the 95% CI was very wide and overlapped. The adjusted HR for past history of HCV infection (anti-HCV-positive but HCV RNA-negative) was 2.6 (95% CI=0.6-11.5) (Table 4).

**Table 4:** Hazard ratios (HRs) and 95% confidence intervals (CIs) of hepatocellular carcinoma (HCC) according to hepatitis C virus infection status and genotype

**Anti-HCV**	**HCV RNA**	**HCV genotype**	**No. of subjects**	**No. of HCC**	**Person-years**	**Incidence (100,000 person-year)**	**HR**^**a**^**(95% CI)**	**HR**^**b**^**(95% CI)**
Negative			6083	31	56,516.3	54.9	1.0 (reference)	1.0 (reference)
Positive	Positive	1	52	8	476.5	1,678.9	28.3 (13.0-61.5)	29.7 (13.6-46.8)
		2	84	1	850.7	117.6	2.1 (0.3-15.4)	2.2 (0.3-16.2)
		1 & 2	6	2	51.1	3,913.9	65.0 (15.5-272.3)	68.7 (16.4-288.4)
	Negative		109	2	1,045.2	191.4	3.4 (0.8-14.4)	2.6 (0.6-11.5)

^a^HR: age- and sex-adjusted. ^b^HR: age-, sex- and HBsAg positivity-adjusted. Two hundred and thirty-six subjects including 2 HCC cases had no information on anti-HCV, and were excluded from the analysis.

The population-attributable fraction of HCC due to HBV and HCV infection were 36.9% (95% CI=20.0-50.2%) and 26.6% (95% CI=11.3-39.2%), respectively, in the study population (data not shown).

## Discussion

The present study provides a comprehensive estimation of HCC risk according to HBV and HCV mono- and coinfection, using data from a community-based prospective cohort study in an area of Korea where HCC incidence is high. HBV is by far the most important risk factor for HCC in Korea. To our knowledge, this is the first study to explore the risk of HCV and discover the synergistic effect of HBV/HCV coinfection for HCC in a prospective cohort study in the general Korean population. Furthermore, in the current study it was evident that HCV genotype 1b increased HCC risk significantly more than genotype 2.

The role of chronic infection with HBV in the etiology of HCC is well established [1,2]. A community-based prospective cohort study titled the Risk Evaluation of Viral Load Elevation and Associated Liver Disease/Cancer-Hepatitis B Virus (REVEAL-HBV) study in Taiwan has reported high serum HBV DNA level, HBV genotype C, precore G1896A mutant and basal core promoter A1762T/G1764A double mutant as predictors of HCC risk [9,27]. The adjusted HR (13.7) of HCC by HBsAg positivity in the present study was compatible with previous study results, including two recent meta-analyses: one that included 37 case–control and 10 cohort studies conducted worldwide [5], and another that used Korean data [15]. In addition, the risk of HCC by HBV infection was around two-fold higher than by HCV infection in present study.

One recent meta-analysis estimated the RR of HCC to be as much as 12-fold higher in people infected with HCV [5]. However, in Korea, there was no report of the RR of HCV, although some case–control studies reported odds ratios for HCV [28-30], and a meta-analysis reported a pooled odds ratio of HCC of 11.5 for anti-HCV-positive individuals [15]. In this respect, the present study contributes more evident data on the effect of HCV infection on HCC risk in Korea to the scientific literature by reporting RRs from a prospective cohort study, although the RR of HCC by anti-HCV positivity was lower than expected.

The synergistic effect of HBV/HCV coinfection on HCC risk (SI=4.5, 95% CI=1.3-15.5) identified in this study was another meaningful finding. Indeed, HBV/HCV coinfection is not uncommon, particularly in countries with a high prevalence of HBV or HCV, as the two viruses share with some modes of transmission [5].

It was not clear if there is a risk difference between HCV genotype 1 and 2 regarding the previous studies in Korean, even though HCV genotype 1b and 2a are dominant. The present study suggested that the HR of HCC was significantly higher in individuals infected with HCV genotype 1 (adjusted HR=29.7, 95% CI=13.6-46.8) than genotype 2 (adjusted HR=2.2, 95% CI=0.3-16.2). This strongly supports the results from a recent meta-analysis, which suggested that subjects infected with HCV genotype 1b had almost double the HCC risk of those infected with other HCV genotypes [8]. Moreover, the present study showed multiplicative increase in HR of HCC in individuals who had mixed infection with HCV genotype 1 and 2 (adjusted HR=68.7, 95% CI=16.4-288.4), compared to HR of solitary genotype 1 or 2 infection. To the best of our knowledge, our study is the first to show this multiplicative relationship. HCV genotype is an important determinant of the virologic response to HCV treatment, whereas differences in disease pathogenesis among genotypes may also exist. Genotype 1 is associated with a more aggressive disease, worse response to therapy, and higher risk of cirrhosis and HCC development [8]. There are limited studies on HCV mixed genotype infection among high-risk individuals [31,32] but pathogenecity of mixed infection and its effect on disease progression and treatment have not yet been elucidated. On the other hand, HBV genotype was not evaluated in the present study as genotype C was predominant (99%) among the HBV genotypes in Koreans [16-18]. In the REVEAL study, HBV genotype C showed higher risk of developing HCC (aRR=1.76; 95% CI=1.19-2.61) and cirrhosis compare to HBV genotype B [33].

There is convincing evidence that alcohol drinking and tobacco smoking increase the risk of primary HCC [34-36] and synergistic effects between hepatitis infection and tobacco smoking or alcohol drinking in the development of HCC has been recently suggested [37-41]. Heavy alcohol drinking significantly elevated HCC risk after adjustment in this study. Tobacco smoking elevated HCC risk in the crude analysis, but was not significant after adjustment. Neither FBS level or obesity were related to HCC risk in this study, although there is growing evidence suggesting that obesity and diabetes mellitus (DM) might be independent risk factors for HCC [20,39,42-45]. There was a substantial amount of missing information on FBS (27% of the study population). However, the HCC risk was not statistically significant (aHR=1.7, 95% CI=0.9-3.4) in the group of unknown FBS. In addition, the HCC risk was not elevated among subjects with DM history, as a proxy of FBS in the independent regression analysis (data not shown). Insignificant result of DM and obesity in risk of HCC development may be due to predominant role of HBV and HCV as a risk factor of HCC in this study population. Dietary ingestion of aflatoxins, one of the risk factor of HCC in developing countries, is very rare in Korea.

The present study offers comprehensive scientific evidence on the effect of HBV and HCV infection, as well as some other potential risk factors, on HCC development. Nevertheless, it has several limitations that should be considered when interpreting its data. Firstly, the size of the case population was not very large (50 HCC cases). In particular, a very small number of HCC cases were identified in each category of HCV genotype, including only 1 HCC case in the group infected with HCV genotype 2. Coinfection with HCV genotype 1 and 2 showed higher HR than genotype 1 alone, but their 95% CI were very wide and overlapping due to small number of HCC cases. However, considering the relatively lower prevalence of HCV infection in Korea, and the results of this community-based long-term prospective follow-up study are valid enough to support a causal relationship. Secondly, the risk factors were investigated at cohort enrollment, with no repeated measure. Thus there were no considerations of updated information for changes in infection status, interventions for liver diseases and/or health behaviors that took place after study recruitment. Thirdly, viral load of HBV and HCV infection was not evaluated in this study. Serum HBV DNA level is a major predictor of HCC development [9]. Patients with a high viral load of HCV respond poorly to interferon therapy [46] and had a significantly higher HCC risk (RR, 2.35; 95% CI, 1.02-5.43) than did those with a low viral load after interferon treatment [47]. However the current study could not provide HCC risk by viral load of HBV and HCV.

Compared to hospital-based case–control studies in Korea (HBsAg 65.4-72.3%; anti-HCV 7.6-19.3%) [28-30], the seroprevalence of HBsAg (39.1%) and anti-HCV (32.6%) among HCC cases in the present study was relatively low. Although the serological evidence of chronic infection with HBV and HCV remains relatively constant over time, there is a possibility that infection status can change between baseline and at diagnosis of HCC. Otherwise occult HBV or HCV infection may exist in HCC cases that are negative for markers of HBV and HCV infection. Recently there has been growing evidence of occult HBV and HCV infection. Occult HBV infection was found in 0.7% of HBsAg-negative individuals in the general adult population in Korea [48]. Although the mechanism and clinical implications have not yet been elucidated, occult HBV infection can also be transmitted and may contribute to the development of HBV-associated diseases such as HCC [49,50].

Nevertheless, this study also has much strength. It is a prospective community-based cohort study that was able to link to a national cancer registry to evaluate the causal relationship of HBV, HCV and other behavioral risk factors with HCC development, while all previous cohort studies reporting the RRs of HCC by viral hepatitis infection have used secondary data (i.e., medical insurance claims) and included no information about HCV infection and related behavioral risk factors (e.g., blood transfusion and acupuncture) [45,51,52]. In addition, the present study provides additional evidence on HCC in an HBV endemic area with comprehensive analyses of HCV infection, especially for different HCV genotypes and coinfection with HBV.

## Conclusions

The present findings add to previous observations suggesting that HBV infection is by far the most important risk factors for HCC in HCC prevalent and HBV endemic areas. Furthermore, investigation of the distribution of different HCV genotypes, as well as the differences in HCC risk by HCV genotype and its coinfection with HBV, may significantly contribute to disease control, or progresses in prevention, such as the development of an effective vaccine, or implementation of an HCC screening program, to improve disease outcome.

## Abbreviations

anti-HBs: Antibodies against HBsAg; anti-HCV: Antibodies against HCV; ALT: Alanine aminotransferase; AST: Aspartate aminotransferase; BMI: Body mass index; CI: Confidence interval; HBsAg: Hepatitis B surface antigen; HBV: Hepatitis B virus; HCV: Hepatitis C virus; HCC: Hepatocellular carcinoma; HR: Hazard ratio; RR: Relative risk; SI: Synergy index.

## Competing interests

The authors declare that they have no competing interests.

## Authors’ contributions

JKO carried out the field survey, conducted the analysis of the data and drafted the manuscript. HRS designed and conducted the study, and drafted and revised the manuscript. MKL participated in conducting the field study and helped to revision the manuscript. HC performed the statistical analysis. DIK carried out the serologic tests. YJ and HY carried out the genotyping. KYY conceived of the study, and participated in its design and coordination. All authors read and approved the final manuscript.

## Pre-publication history

The pre-publication history for this paper can be accessed here:

http://www.biomedcentral.com/1471-2407/12/452/prepub

## Supplementary Material

Additional file 1 **Table S1.** Distribution of hepatitis C virus (HCV) genotypes among 142 HCV RNA-positive subjects in the study population. Click here for file
