# Supplementary material for: Multiplicative synergistic risk of hepatocellular carcinoma development among hepatitis B and C co-infected subjects in HBV endemic area: a community-based cohort study
Source: BMC Cancer. 2012 Oct 5;12:452. doi: 10.1186/1471-2407-12-452 (PMC3520797; doi:10.1186/1471-2407-12-452)
Supplement: Additional file 1 — Table S1. Distribution of hepatitis C virus (HCV) genotypes among 142 HCV RNA-positive subjects in the study population. [file 1471-2407-12-452-S1.doc]

Supplement table 1. Distribution of hepatitis C virus (HCV) genotypes among 142 HCV RNA-positive subjects in the study population

| HCV genotypes | Subtypes | No. of subjects |
| --- | --- | --- |
| 1 | 1 | 2 |
|  | 1a | 3 |
|  | 1b | 47 |
| 2 | 2 | 1 |
|  | 2a | 39 |
|  | 2a/2c | 44 |
| 1 and 2 | 1 and 2a/2c | 2 |
|  | 1b and 2a/2c | 3 |
|  | 1b and 2a | 1 |
| Total |  | 142 |
